# Mycotoxin Notifications in the European Rapid Alert System for Food and Feed (RASFF): Temporal Trends and Implications for Food Safety and Exposure

**DOI:** 10.3390/toxins18090381

**Published:** 2026-09-04

**Authors:** Sandra Juárez-García, Ana Isabel Fernández-Díez, Rafael Mora-Medina, Nahúm Ayala-Soldado, Antonio Lora-Benitez, Rosario Moyano-Salvago, Ana María Molina-López

**Affiliations:** 1 Departamento Anatomía y Anatomía Patológica Comparadas y Toxicología, Unidad de Investigación Competitiva Zoonosis y Enfermedades Emergentes desde la Perspectiva de Una Salud ENZOEM, Universidad de Córdoba, Campus de Rabanales, Edificio Darwin, 14071 Córdoba, Spain; sandra1999.sjg@gmail.com (S.J.-G.); ft1fedia@uco.es (A.I.F.-D.); v02momer@uco.es (R.M.-M.); v22ayson@uco.es (N.A.-S.); v12lobea@uco.es (A.L.-B.); ft1mosam@uco.es (R.M.-S.); 2 Instituto de Agricultura Sostenible (IAS-CSIC), 14004 Córdoba, Spain

**Keywords:** mycotoxins, aflatoxins, ochratoxins, RASFF, food safety, public health

## Abstract

Mycotoxin contamination in food and feed remains a major public health concern because of its potential toxicological impact and its global economic implications. Aflatoxins and ochratoxin A are of particular concern due to their carcinogenic, hepatotoxic, and nephrotoxic effects, highlighting the need for effective surveillance systems to reduce dietary exposure in the population. This study analyzes 2479 mycotoxin-related notifications concerning both food and feed reported through the iRASFF system during the period 2020–2024, with the aim of characterizing temporal notification trends, identifying the most affected food products and geographical regions, and evaluating the evolution of control strategies implemented by competent authorities. The results did not show a statistically significant long-term increasing trend in the number of mycotoxin notifications, although interannual fluctuations may reflect both changes in contamination patterns and improvements in detection and reporting systems. Products originating outside the European Union accounted for 90.44% of notifications and showed a significantly higher mean number of notifications than products originating within the European Union. Turkey (24%) and the United States (14%) were the most frequently reported countries of origin at a global level. Aflatoxins were the most frequently detected mycotoxins in food (84%), followed by ochratoxin A (15%), mainly affecting nuts, cereals, fruits, vegetables and herbs and spices. These findings demonstrate that mycotoxins continue to represent a persistent challenge for European food safety. Strengthening surveillance systems, enhancing international cooperation, and adapting control strategies to emerging environmental and trade-related factors are essential to minimize consumer exposure, particularly to compounds with genotoxic and carcinogenic potential, and to improve risk management within the food supply chain.

## 1. Introduction

Food safety constitutes one of the fundamental pillars of public health and the sustainability of agri-food systems [1]. In recent decades, the globalization of markets, agricultural intensification, and climate change have increased the complexity of supply chains, favoring the emergence and dissemination of chemical, biological, and physical contaminants. Among these, mycotoxins stand out as natural xenobiotics of high toxicological relevance due to their ability to interact with biological systems, as well as their significant importance not only from a health perspective but also in economic and regulatory terms [2].

Mycotoxins are mainly produced by filamentous fungi belonging to the genera *Aspergillus*, *Penicillium*, and *Fusarium*, and can develop in a wide variety of agricultural commodities and processed products, both during cultivation and throughout storage or transport [3]. The most frequently affected food matrices include cereals, nuts, dried fruits, spices, compound feed, and milk [4,5]. From a toxicological perspective, certain mycotoxins are of particular concern due to their genotoxic and carcinogenic properties. Aflatoxin B1 (AFB1) has been classified by the International Agency for Research on Cancer (IARC) as a Group 1 carcinogen in humans, whereas ochratoxin A (OTA) is associated with nephrotoxic effects and has been classified as possibly carcinogenic to humans (Group 2B) [6,7].

The control of mycotoxins represents a major challenge due to their ubiquity and stability, as these compounds are resistant to conventional technological treatments and may persist after food processing [8]. This persistence increases the likelihood of chronic dietary exposure at low concentrations, a key aspect in toxicological risk assessment. In addition, climate change is modifying the environmental conditions that favor the development of mycotoxigenic fungi, expanding their geographical distribution and increasing the risk of contamination, particularly in Mediterranean regions [9,10].

The impact of these toxins on public health is direct, through the consumption of contaminated food, and indirect, via products of animal origin derived from animals fed with contaminated feed. A well-established example of this carry-over pathway is the biotransformation of AFB1 ingested through contaminated feed into aflatoxin M1 (AFM1) in lactating animals, followed by its excretion in milk and the consequent potential for human dietary exposure [4,5]. Chronic exposure has been associated with hepatotoxic, nephrotoxic, teratogenic, and immunosuppressive effects, particularly affecting vulnerable populations such as infants, children, pregnant women, and immunocompromised individuals [11]. In animals, AFB1 undergoes hepatic biotransformation into different metabolites and can exert marked immunotoxic effects, affecting both innate and adaptive immune responses and increasing susceptibility to disease [12]. In this context, both the World Health Organization (WHO) and the European Food Safety Authority (EFSA) agree that, for genotoxic mycotoxins such as aflatoxins, no safe level of intake can be established [13,14], reinforcing the need to minimize their presence in the food chain.

Risk assessment requires an integrated approach that considers the entire supply chain, in line with the One Health framework, which recognizes the interdependence between human, animal, and environmental health [14]. This perspective is particularly relevant for mycotoxins, as environmental conditions influence fungal growth and toxin production, contaminated feed can affect animal health and productivity, and certain mycotoxins or their metabolites may subsequently enter the human food chain through animal-derived products. In this context, the systematic monitoring of mycotoxins plays a key role both from regulatory and toxicological perspectives, enabling the identification of exposure patterns potentially relevant to the population [2].

Within the European Union, the Rapid Alert System for Food and Feed (RASFF) constitutes the main tool for coordination and rapid communication between Member States in response to food safety risks, enabling the immediate notification of incidents and the implementation of measures such as the withdrawal of contaminated products. Previous analyses of RASFF notifications have highlighted the predominance of aflatoxins and identified temporal trends, notification types, food categories, and countries involved in mycotoxin-related alerts [15]. The system is currently regulated by Commission Implementing Regulation (EU) 2019/1715 of 30 September 2019 [16], which establishes the rules for the operation of the Information Management System for Official Controls (IMSOC).

Preventive approaches must be based on integrated strategies combining analytical monitoring, good agricultural and storage practices, and the training of food business operators [17]. In addition, European policies aimed at reducing dietary exposure, such as the establishment of maximum levels under Commission Regulation (EU) 2023/915 [18] (regulations applicable during the study period), reinforce risk management. The persistence and interannual variability of mycotoxin-related notifications, together with evolving climatic conditions, international trade patterns, and regulatory surveillance, highlight the need for an updated analysis of their distribution and characteristics during the 2020–2024 period [10,19,20].

Previous RASFF-based studies have generally focused on specific mycotoxins, commodities, countries, or earlier time periods, and an integrated analysis of mycotoxin-related notifications covering the complete 2020–2024 period using data retrieved directly from the iRASFF platform, which became operational in 2020, remains limited. Therefore, the aim of this study is to analyze the evolution of iRASFF notifications related to the presence of mycotoxins in food and feed during the period 2020–2024, identifying the most frequently reported mycotoxins, the food matrices involved, the countries of origin and notifying countries, as well as patterns in notification categories, risk decisions, and control measures within the European rapid alert system. This analysis provides an interpretation from the perspective of food-safety surveillance, potential population exposure, and the toxicological relevance of these xenobiotics within the food chain.

## 2. Results and Discussion

### 2.1. European Evolution of the Number of Notifications

During the period 2020–2024, a total of 2479 notifications related to the presence of mycotoxins in food and feed were recorded in the RASFF database. These notifications included alerts, border rejections, and information notifications, forming the basis of the present analysis.

#### 2.1.1. Historical Evolution of the Number of Notifications (2012–2024)

**Figure 1:**
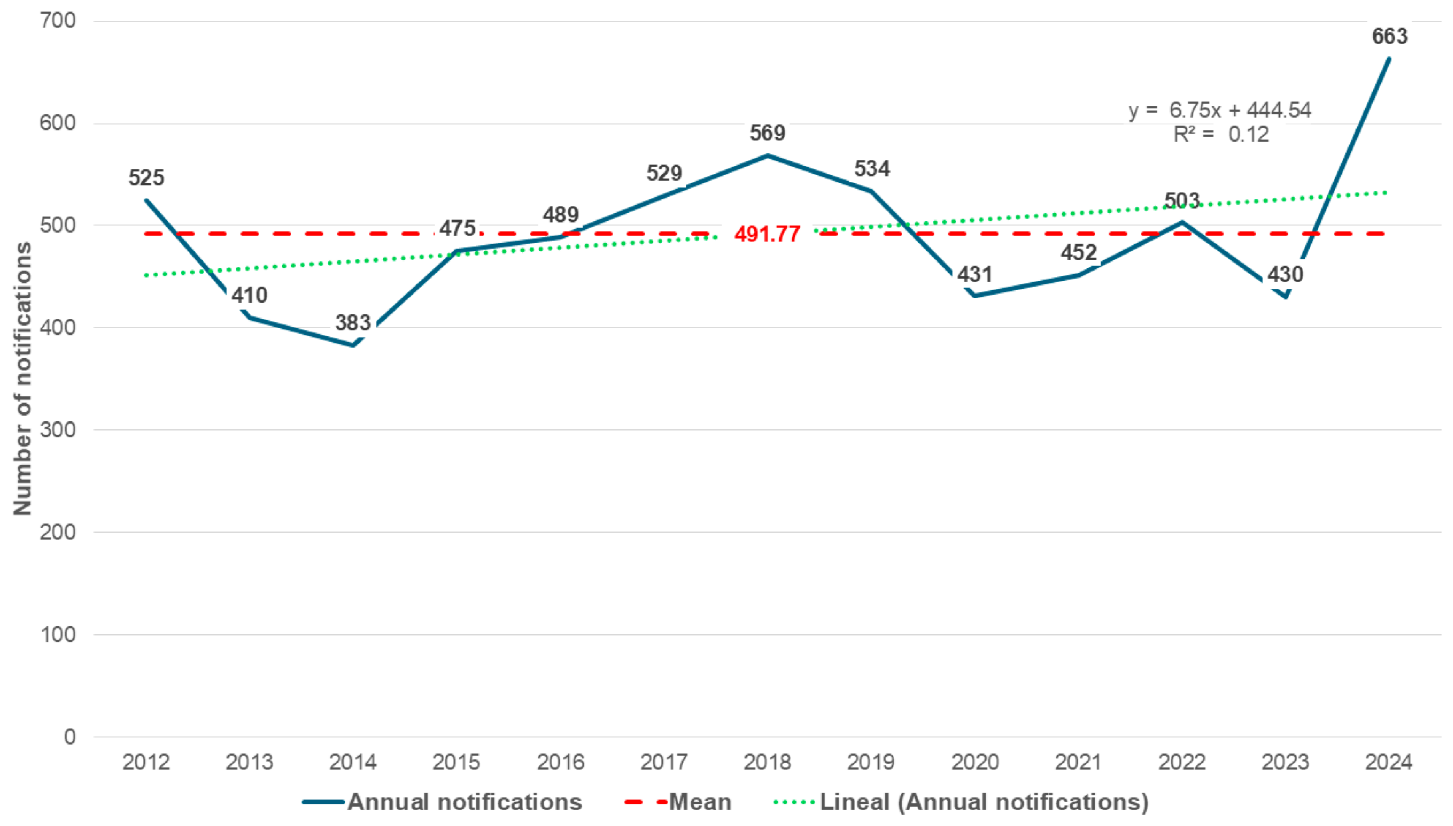
Evolution of the incidence of mycotoxin notifications in the European Union (2012–2024).

To contextualize these findings within a broader temporal framework, the historical evolution of mycotoxin notifications in the European Union was analyzed for the period 2012–2024, based on RASFF annual reports (Figure 1). The inclusion of this extended time series provides continuity with previous studies and allows recent notification patterns (2020–2024) to be interpreted within a longer-term surveillance context [15,21,22]. Over the study period, the annual number of notifications showed considerable interannual variability, with a median of 489 notifications per year (range: 383–663). The highest value was recorded in 2024 (663 notifications), which may reflect improvements in detection capacity and the strengthening of official control systems for chemical hazards of public health relevance.

Following a decline between 2012 and 2014, notification frequencies increased until 2018, after which marked year-to-year fluctuations were observed. The lowest values after 2015 occurred in 2020 (431 notifications) and 2023 (430 notifications), whereas 2024 recorded the highest number of notifications throughout the study period.

The relatively low number of notifications recorded in 2020 coincided with the onset of the COVID-19 pandemic. In that year, border rejection notifications decreased by approximately 30% compared to the previous year. This reduction may be associated with temporary disruptions in international trade and control activities during the pandemic period, which affected the agri-food sector in the European Union [23,24,25]. Subsequent years show a progressive recovery in agri-food trade and control systems as supply chains stabilized and monitoring activities were progressively restored. From 2020 onwards, the trend remained irregular, although a marked increase was observed in 2024. This pattern may be influenced by multiple factors, including changes in trade flows, surveillance intensity, and analytical capacity; climatic and environmental conditions may influence the ecology of mycotoxigenic fungi and consequently the likelihood of toxin production.

Importantly, mycotoxigenic fungi differ in their ecological requirements and in the stage of the production chain at which contamination most commonly occurs. *Fusarium* species are predominantly associated with pre-harvest infection of crops and are strongly influenced by field conditions during crop development, whereas *Aspergillus* and *Penicillium* species are more frequently associated with post-harvest contamination and storage, although overlap between these ecological niches may occur. Temperature, water activity, crop moisture, and storage conditions therefore influence fungal growth and mycotoxin accumulation differently depending on the fungal genus and commodity involved. Consequently, changes in climatic conditions may alter both the geographical distribution of mycotoxigenic fungi and the relative importance of pre-harvest and post-harvest contamination pathways. This distinction is important because warmer conditions may favor thermotolerant species such as *Aspergillus*, whereas other genera may respond differently to temperature and moisture regimes [26,27,28].

To evaluate the temporal evolution of annual mycotoxin notifications, a simple linear regression analysis was performed using year as the independent variable. Although the regression slope was positive (6.75 notifications per year), suggesting an apparent increase over time, the relationship was not statistically significant (R^2^ = 0.123, F = 1.54, *p* = 0.241). These findings indicate that the substantial interannual variability observed throughout the study period precludes the identification of a consistent long-term linear trend.

Furthermore, as noted by Kowalska and Manning [21], RASFF notifications reflect surveillance intensity and reporting practices rather than actual mycotoxin prevalence or population exposure. Consequently, these trends remain inherently influenced by shifting control strategies, sampling efforts, and international trade flows.

Overall, although annual notification frequencies exhibited marked fluctuations and reached their highest level in 2024, no statistically significant long-term increasing trend was observed during the period 2012–2024. Nevertheless, the sustained magnitude of notifications highlights the need to maintain risk assessment strategies aimed at minimizing chronic dietary exposure to mycotoxins, particularly in vulnerable population groups.

It is noteworthy that mycotoxins represent one of the most frequently reported hazards in certain plant-based matrices, such as herbs and spices, maintaining a consistent presence over time [22]. This recurrence in commonly consumed products reinforces the importance of integrating notification data into dietary exposure assessment models and toxicological risk prioritization frameworks.

#### 2.1.2. Distribution and Temporal Evolution of Mycotoxin Notifications (2020–2024)

The annual distribution of notifications associated with different mycotoxins (2020–2024) shows a clear predominance of aflatoxins, which accounted for 2076 notifications (84% of the total), representing most reported cases. Annual frequencies ranged from 335 notifications in 2023 (minimum) to 540 in 2024 (maximum), with a median of 393 notifications per year. This pattern reflects both the widespread occurrence of these mycotoxins in the food chain and their priority relevance within surveillance and control systems, in agreement with Owolabi et al. [20], who identified aflatoxins as the main cause of RASFF notifications over a ten-year period, particularly in nuts. From a toxicological perspective, this high frequency is particularly relevant given that aflatoxins are classified as Group 1 carcinogens by IARC, and chronic exposure, even at low concentrations, has been associated with an increased risk of hepatocellular carcinoma [6].

Ochratoxins ranked second, with 361 notifications (15%), showing an increasing trend (from 44 cases in 2020 to 117 in 2024). This increase may reflect both a higher number of notifications and/or improvements in detection capacity, potentially influenced by climatic factors that favour fungal proliferation [10,19]. Given their association with nephrotoxicity and possible carcinogenicity, this trend is of relevance in the context of chronic dietary exposure.

From a temporal perspective, the data indicate an increase in total notifications in 2024, whereas 2023 showed the lowest number of notifications, mainly due to a reduction in aflatoxin-related cases. To evaluate temporal variation in the distribution of mycotoxin notifications, a chi-square (χ^2^) test of independence (year × type of mycotoxin) was performed. Although the associations were statistically significant (*p* < 0.05), the Cramér’s V value indicated a very weak effect size (V = 0.07). Therefore, the observed differences should be interpreted with caution, as the statistical significance may partly reflect the large sample size rather than a substantively meaningful association. This suggests that the relative distribution of mycotoxins remained largely stable over time despite fluctuations in absolute notification counts. In addition, a chi-square (χ^2^) goodness-of-fit test showed a significant deviation from a uniform distribution (*p* < 0.001), confirming a clear disproportionality in notification frequencies, with aflatoxins being the main contributor to this pattern. Overall, this distribution indicates that the RASFF system is largely driven by a limited number of high-priority mycotoxins, supporting the need to focus surveillance and control strategies on these compounds.

#### 2.1.3. Proportional Trend of Mycotoxins in the Global Alert Landscape

**Figure 2:**
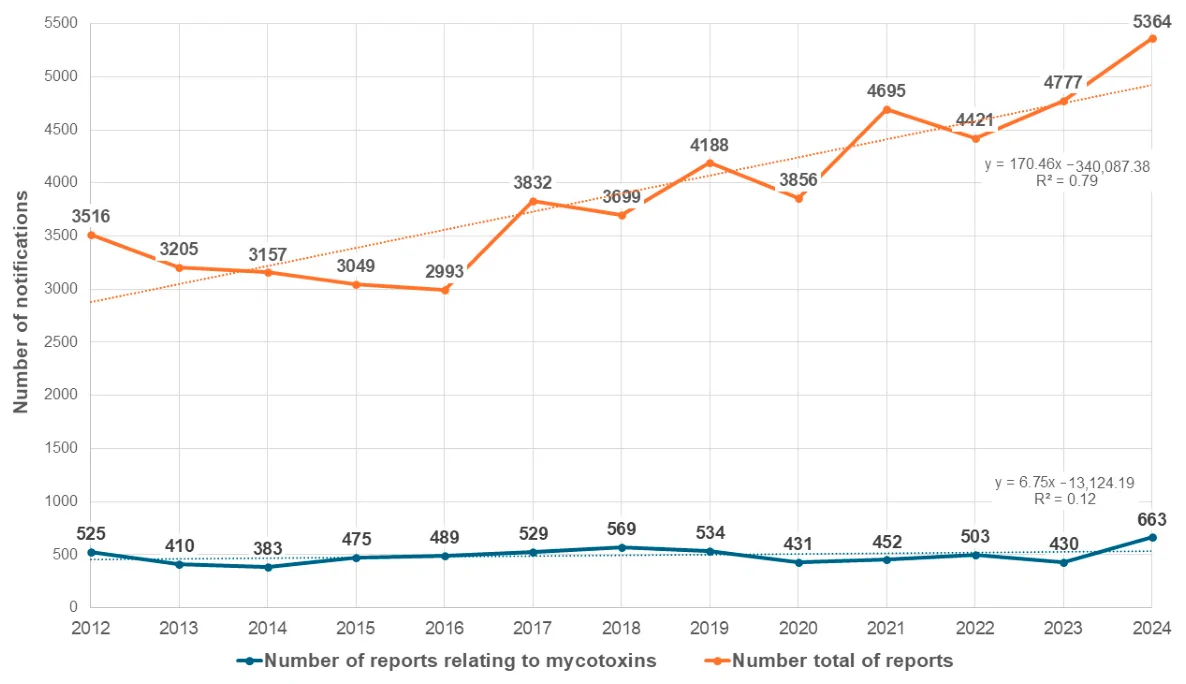
Evolution of total RASFF notifications and mycotoxin notifications (2012–2024).

To evaluate whether the observed trends are specific to mycotoxins, their evolution was compared against the total volume of RASFF notifications (Figure 2). While total notifications in the RASFF system have shown a consistent and robust increase—rising from 3516 in 2012 to 5364 in 2024—the notifications specifically linked to mycotoxins have remained remarkably stable, fluctuating between 400 and 600 annual cases. Consequently, the relative importance of mycotoxins within the system has undergone a significant decline, dropping from representing 15–16% of total notifications in the 2015–2018 period to 9–12% in the most recent years (2023–2024).

### 2.2. Distribution of Notifications by Notifying Country (2020–2024)

Of the total 2479 notifications related to mycotoxin contamination during the study period (2020–2024), 2375 (96%) were reported by European Union Member States, while 4% were notified by non-EU countries. The non-EU countries reporting notifications were the United Kingdom (50), Switzerland (47), and Norway (7), which likely reflects their integration into European trade networks and cooperation with the RASFF system rather than a higher intrinsic incidence of mycotoxin contamination.

**Figure 3:**
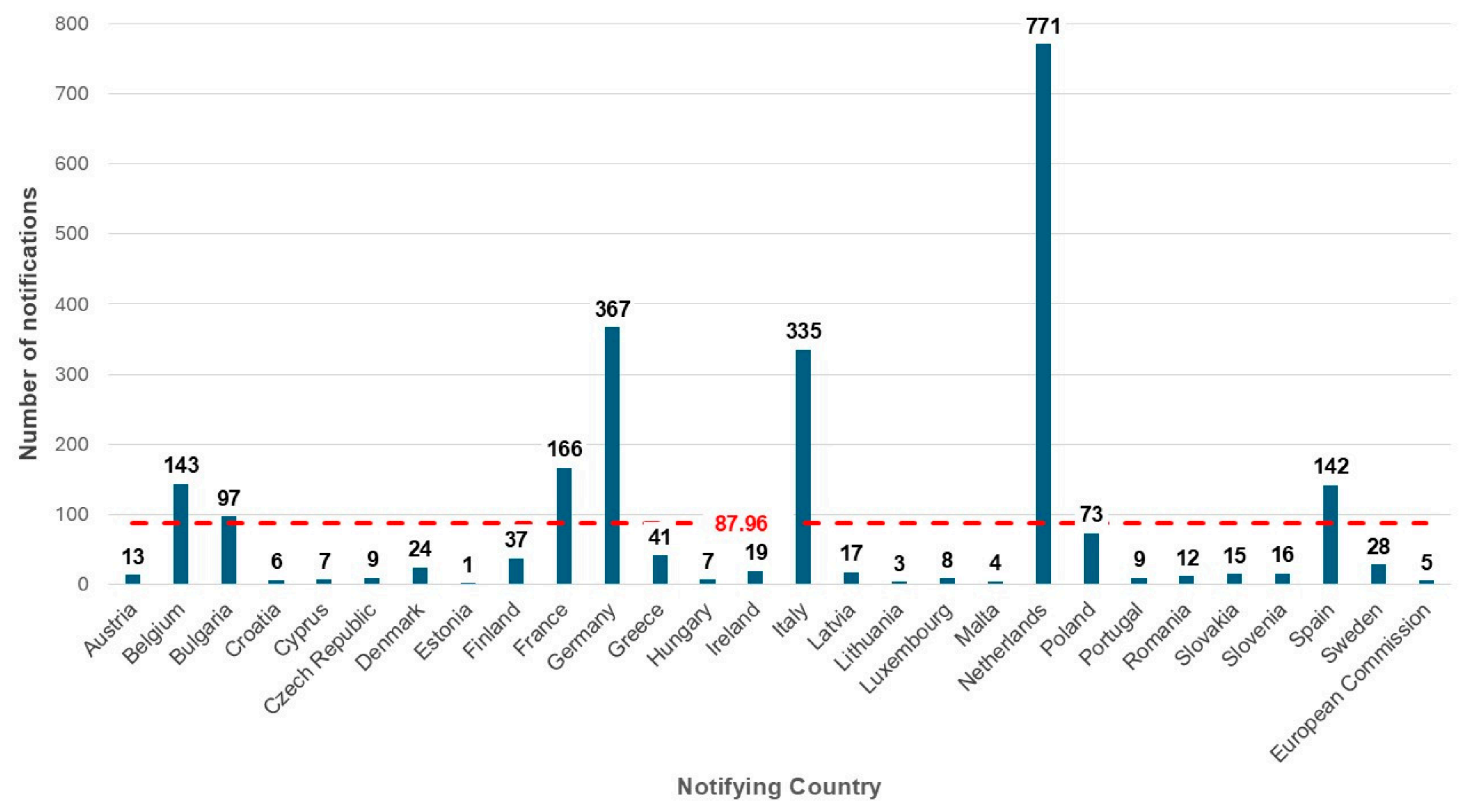
Notifying countries within the European Union (2020–2024).

The number of notifications reported by EU Member States showed marked heterogeneity, ranging from 1 to 771 notifications (Figure 3) with a median of 16.5 notifications. This wide range reflects a highly uneven distribution among countries. The Netherlands, Germany, and Italy reported the highest number of notifications (771, 367, and 355, respectively). These differences may be associated with their role as major entry points for imported goods, high volumes of international trade, and the intensity of official control activities.

In contrast, Estonia and Lithuania reported the lowest number of notifications, which may be related to lower import volumes, reduced transit of high-risk agri-food products, or differences in national control and trade patterns. Spain reported 142 notifications, exceeding the overall mean (88). This finding should be interpreted with caution and may reflect Spain’s role as an important entry point for agri-food products originating from regions with higher mycotoxin occurrence, as well as its analytical capacity and inspection intensity.

Importantly, the number of notifications should not be interpreted directly as an indicator of higher internal risk, but rather as a reflection of inspection activity, trade dynamics, and logistical positioning within the European food supply chain. It should be emphasized that the notifying country is not necessarily the country of origin of the affected product. High numbers of notifications issued by some EU Member States, particularly major commercial and logistical hubs, may reflect greater import volumes, inspection intensity, border-control activity, and redistribution through their ports rather than deficiencies in their domestic food systems. The concentration of notifications in specific Member States highlights the importance of considering not only contamination patterns but also trade flows and critical entry points in the overall assessment of food safety risk and potential population exposure. These findings further suggest that notification frequency is influenced by structural characteristics of national control systems and trade logistics within the EU, which may contribute to differential patterns of dietary exposure across populations.

### 2.3. Origin of Notifications (2020–2024)

A total of 90.44% of mycotoxin-related notifications recorded in the iRASFF system were associated with products originating from non-European Union (non-EU) countries (Table 1). This finding may be influenced by the strict EU regulatory framework regarding maximum levels of mycotoxins, as well as by the intensity of official controls at border entry points, although it may also reflect differences in production systems and regulatory approaches across regions. In addition, the lack of full regulatory harmonization between countries may contribute to this pattern, as non-EU countries may operate under different food safety standards and monitoring programmes compared to those established within the EU.

Environmental conditions may also play a relevant role, as climatic factors such as temperature, humidity, and insect activity influence the growth of mycotoxigenic fungi and the production of mycotoxins. Several studies have reported that climate change may contribute to an increased incidence of mycotoxins through its effects on fungal ecology [10,28,29,30]. Similarly, an increase in the occurrence of mycotoxins in food over the past two decades has been documented, potentially associated with changing climatic conditions. Aflatoxin-producing fungi (mainly *Aspergillus flavus* and *A. parasiticus*) have expanded in warm, tropical, and subtropical regions, contributing to increased cases of contamination in the food chain, including the presence of AFM1 in milk [10,19]. These findings highlight the need for integrated prevention strategies across primary production and post-harvest stages to limit fungal growth and mycotoxin production.

From a population exposure perspective, the high proportion of notifications associated with non-EU products underscores the importance of border controls as a preventive barrier against the entry of contaminants of toxicological relevance, particularly those with carcinogenic and nephrotoxic potential, into the European market.

The analysis of data collected between January 2020 and December 2024 shows a highly uneven distribution of notifications across countries, ranging from 1 to 585 notifications with a median of 5 notifications per country. The inclusion of relative percentages in Table 1 facilitates a clearer visualization of the concentration of notifications in specific countries, with Turkey and the United States emerging as the main contributors among non-EU countries.

At the global level, considering both EU and non-EU countries, Turkey (24%) and the United States (14%) were the most frequently reported countries of origin of mycotoxin-related notifications during the study period. These results are consistent with the distribution observed among non-EU countries, where both countries also ranked among the main contributors, although with higher relative proportions when calculated exclusively within that group. Turkey’s predominance as the primary country of origin aligns with the analysis by Eissa et al. [31]; their study of RASFF notifications concerning organic products also identifies Turkey as the leading country of origin for mycotoxin-related notifications, thereby reinforcing the recurrent prominence of Turkish-origin products in mycotoxin-related RASFF notifications. Furthermore, our findings are consistent with those reported by Owolabi et al. [20], who indicated that countries with higher production of food commodities susceptible to mycotoxin contamination tend to report a greater number of notifications, suggesting that climatic conditions and post-harvest handling practices may play a relevant role in shaping notification patterns. The wide diversity of countries associated with mycotoxin notifications highlights that this is a global issue. In addition, 41 notifications were recorded with an unknown country of origin, which may reflect limitations in traceability systems or incomplete data reporting in certain cases. Traceability constitutes a fundamental principle under Regulation (EC) No 178/2002 of the European Parliament and of the Council (article 18) [32], which requires the identification of origin at all stages of the food chain, emphasizing the importance of ensuring transparency and effective control within international food trade.

The country associated with the highest number of notifications was Turkey, accounting for 26% of all non-EU notifications recorded during the study period (Table 1). This was followed by the United States (16%) and India (8%). This pattern may be influenced by a combination of factors, including climatic conditions favourable to the growth of mycotoxigenic fungi, the high production and export of susceptible food commodities, and differences in regulatory limits and control intensity applied in international trade.

These findings underscore that border surveillance constitutes a key component of the European food safety system, acting as a preventive barrier against the introduction of contaminants of toxicological relevance, particularly those with carcinogenic and nephrotoxic potential. Furthermore, they highlight the importance of integrating notification data into exposure-oriented surveillance frameworks and risk prioritization strategies, as well as their value as indirect indicators of population exposure to natural xenobiotics within the context of global food trade.

#### 2.3.1. Incidence of Notifications Originating in the European Union

**Figure 4:**
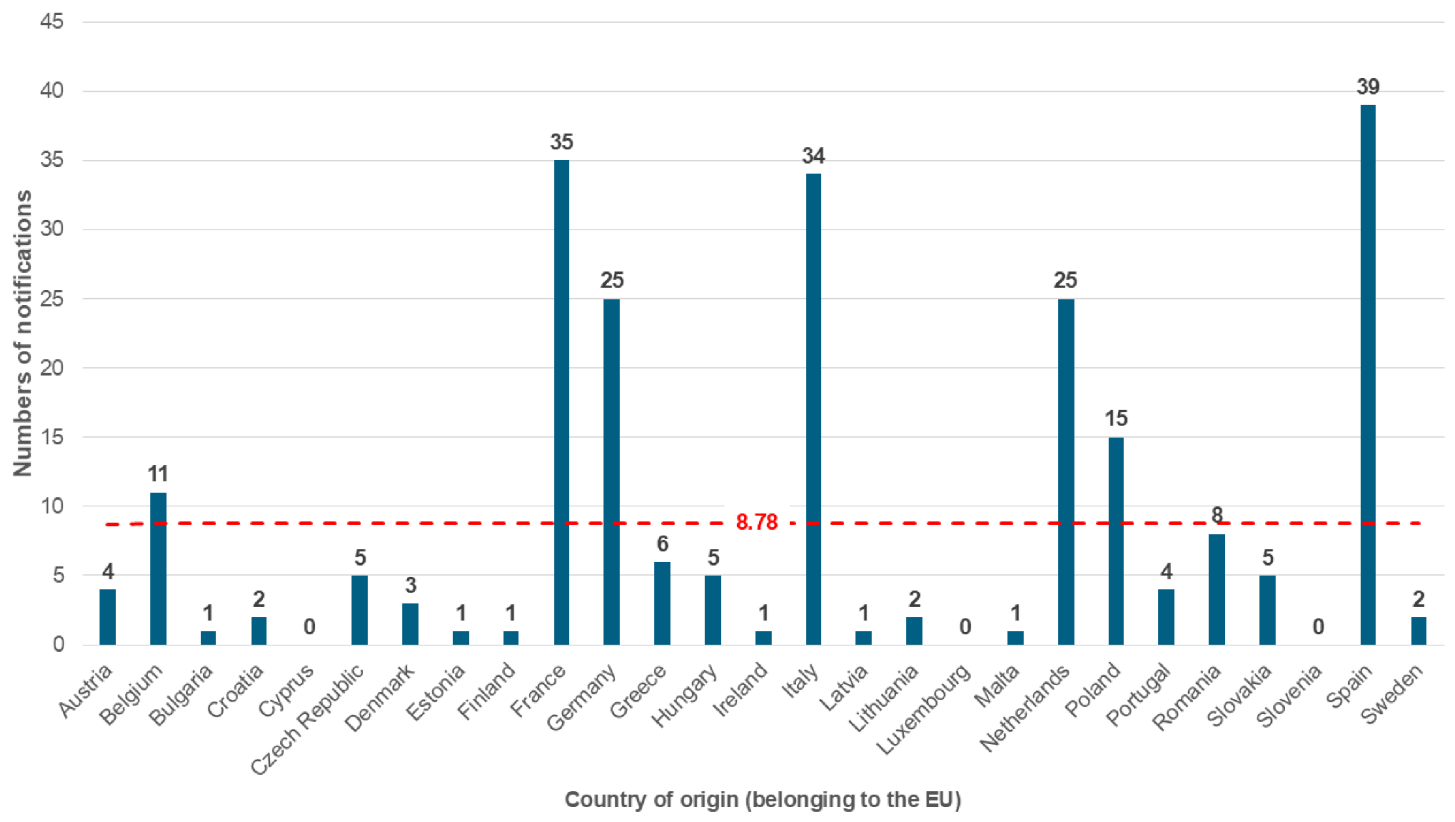
Representation of the number of notifications reported by European Union Member States (2020–2024).

When analyzing the number of mycotoxin notifications by country of origin within the European Union (Figure 4), the mean number of notifications per country was 8.78 ± 11.64, indicating a heterogeneous distribution among Member States. In addition, the median number of notifications per country was 4, with values ranging from 0 to 39 notifications. Spain reported the highest number of notifications (39), followed by France (35), Italy (34), Germany (25), and the Netherlands (25). At the opposite end, Cyprus, Slovenia and Luxembourg reported no notifications, while Bulgaria, Estonia, Finland, Ireland, Latvia and Malta recorded one notification each over the five-year study period. These differences may be associated with factors such as production volume, trade flows, climatic conditions, and the intensity of official controls, although the present study does not allow the establishment of direct causal relationships.

Of the 39 notifications originating from Spain, 51% were related to aflatoxins, 41% to OTA, and 5% to patulin, while the remaining notification corresponded to patulin (2 notifications) and *Claviceps purpurea* (1 notification).

Furthermore, the proportion of OTA notifications in Spain (41%) is higher than the overall proportion of OTA notifications (14%); however, this comparison should be interpreted with caution due to differences in sample size and denominator structure. Several studies have shown that climate change may favour the development of mycotoxigenic fungi under previously less favourable conditions, altering both their geographical distribution and mycotoxin production dynamics, particularly in sensitive crops such as grapes, cereals and nuts [10,19].

Spain is one of the main producers and exporters of fruits, vegetables, and nuts within the European Union, which may contribute to increased detection probability within official control systems, rather than necessarily reflecting a higher intrinsic contamination risk [33]. This aspect is relevant when interpreting potential differences in dietary exposure to mycotoxins linked to production and distribution patterns within the EU.

#### 2.3.2. Distribution of Mycotoxins According to Country of Origin

The profile of mycotoxin notifications varied considerably depending on the geographical origin of the products. Among products originating from non-EU countries, aflatoxins were predominant, accounting for 87.92% of notifications, followed by OTA (12.84%), while other mycotoxins appeared in less than 1% of the notifications.

In contrast, products from EU Member States showed a more heterogeneous mycotoxin profile. Aflatoxins remained the most frequently notified mycotoxin group (42.80%), followed by OTA (30.93%) and ergot alkaloids (11.02%), while patulin (6.78%), fumonisins (5.08%), deoxynivalenol (2.54%), T-2 toxin (1.69%), and zearalenone (0.42%) represented smaller proportions. Because three EU notifications involved mixtures of different mycotoxins, the percentages do not sum to 100%. These notifications were counted only once in the overall total to avoid double counting but were included in each corresponding individual mycotoxin category.

**Figure 5:**
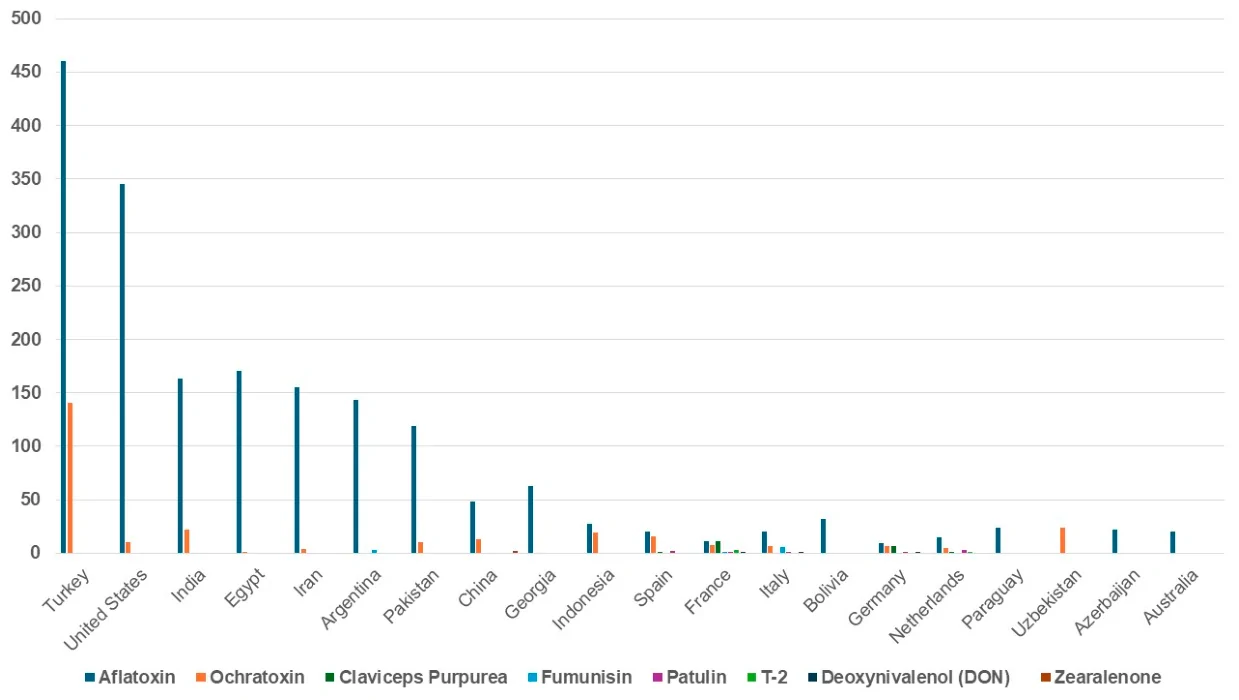
Distribution of mycotoxin notifications among the 15 countries with the highest number of notifications.

At the country level, the distribution of mycotoxin notifications also showed marked geographical differences. Figure 5 presents the mycotoxin profile of the main countries of origin, allowing the relative contribution of the different mycotoxin groups within each country to be visualized.

These differences may partly reflect variations in climatic conditions, agricultural practices, and post-harvest management among different geographical regions. Mycotoxin contamination may occur at different stages of the food chain, from pre-harvest crop production through harvesting, handling, storage and processing. Mycotoxins associated with the genus *Fusarium* such as fumonisins and deoxynivalenol are predominantly associated with pre-harvest infection and field conditions, although contamination and further toxin accumulation may also occur after harvest depending on handling and storage practices. Aflatoxins and OTA may occur under both pre- and post-harvest conditions, although inadequate drying and unfavourable moisture, temperature and storage conditions can play an important role in post-harvest fungal growth and toxin accumulation [26,27,28].

Therefore, the predominance of aflatoxin notifications concerning products from non-EU countries could be consistent with differences in environmental conditions and/or postharvest practices that favour aflatoxin production. However, these results should be interpreted with caution, as notification data do not provide direct information on the specific stage at which contamination occurred or on the agricultural, harvesting, or postharvest practices applied. The greater diversity of mycotoxins observed among EU products, including DON, fumonisins, and T-2 toxin, could suggest a greater contribution of mycotoxins associated with field contamination, although a causal relationship cannot be established from this dataset.

A notable finding was the geographical restriction of ergot alkaloid notifications to products originating from EU Member States. Similarly, deoxynivalenol notifications were associated almost exclusively with products from EU countries, with Ukraine being the only non-EU country of origin associated with a DON notification.

Among the 15 countries with the highest number of notifications, only five were EU Member States (Spain, France, Italy, Germany and the Netherlands), and none of them ranked among the top 10. Aflatoxins were the predominant mycotoxin group in most of the countries, with particularly high numbers of notifications associated with products originating from Turkey (460), the United States (345), Egypt (171), India (163), Iran (155), Argentina (143), and Pakistan (119). OTA was the second most frequently reported mycotoxin in several countries, particularly Turkey (141), India (22), Indonesia (19), Spain (16), and China (13). Most countries showed a clear predominance of aflatoxins; however, France presented a more diverse profile, with an equal number of notifications for aflatoxins and ergot alkaloids associated with *Claviceps purpurea*, as well as notifications for T-2, DON, patulin, fumonisins, and OTA. Italy also showed a relatively diverse profile, with notifications for aflatoxins (20), OTA (7), fumonisins (6), DON (1), and patulin (1). Overall, these results highlight the strong contribution of aflatoxins to the total number of notifications among the 15 countries, while ochratoxin and other mycotoxins contributed to more diverse notification profiles in some countries.

### 2.4. Incidence by Type of Notification

During the study period (2020–2024), 68% of mycotoxin-related notifications corresponded to border rejections, followed by alert notifications (16%), information notifications for attention (13%), and information notifications for follow-up (2%). In addition, a single case of non-compliance was recorded.

The predominance of border rejections indicates that a substantial proportion of detections occur prior to entry into the European market, highlighting the role of border control as a primary barrier to consumer exposure within the operational framework of the RASFF system. This pattern partially differs from the findings reported by Owolabi et al. [20], who observed that 91% of notifications related to mycotoxins in nuts corresponded to border rejections during the period 2011–2021, suggesting that the distribution of notification types may vary depending on the food matrix considered. The presence of alert and information notifications indicates that mycotoxin detection also occurs at later stages of the food supply chain, underscoring the need for continuous monitoring throughout production and distribution systems [22]. Overall, this distribution highlights the importance of integrating preventive controls at border entry points with internal monitoring mechanisms to minimize population exposure to mycotoxins of toxicological relevance across the entire food chain, particularly in the case of contaminants with carcinogenic and nephrotoxic properties.

### 2.5. Incidence by Type of Mycotoxin

Aflatoxins represented the main cause of notifications in the iRASFF system. Within this group, AFB1 was the most frequently notified mycotoxin in iRASFF, with 1848 notifications recorded during the study period. This value reflects notification frequency within the iRASFF surveillance system. The highest incidence was observed in nuts, nut-derived products, and seeds (64%), which are particularly susceptible to fungal contamination during cultivation and storage. This finding agrees with previous studies reporting the high prevalence of aflatoxins and their relevance in food safety within the European Union [34]. Recently, Kow et al. [35] analysed aflatoxin notifications reported through the RASFF between 2010 and 2023, confirming the persistence of aflatoxins as a major food safety concern in the EU and highlighting nuts and nut-derived products among the commodities most frequently associated with notifications. These findings are consistent with those observed in the present study. AFM1 was reported in three notifications and, although limited in number, this finding is of toxicological and public health relevance due to its occurrence in milk, a widely consumed food, especially among vulnerable populations such as infants. In addition, cases of co-contamination were identified, with the aflatoxin–OTA combination being the most frequent (41 cases). This co-occurrence raises considerations regarding potential simultaneous exposure and possible additive or synergistic effects, although such interactions cannot be assessed within the scope of the present study. The notification frequency of aflatoxins observed in this study is consistent with previous analyses of RASFF reports, which identified aflatoxins as the most frequently reported mycotoxins since the 1980s [15].

Ochratoxin A increased over the study period, from 44 cases in 2020 to 117 in 2024. The highest proportion of notifications was observed in fruits and vegetables (52%), followed by cereals and spices. Given that these food categories are commonly consumed, their recurrent involvement in OTA notifications is relevant when considering the potential for chronic dietary exposure and risk assessment [17]. This distribution is consistent with occurrence studies reporting OTA contamination in a wide range of food commodities, particularly cereals, dried fruits, and spices, with contamination patterns influenced by geographical, climatic, and storage-related factors [36]. In addition, the potential health implications of OTA exposure in animals further support the need for continued monitoring and mitigation. Recent experimental evidence in broiler chickens showed that OTA exposure can adversely affect hepatic and renal function and alter immune responses, while dietary supplementation with doum fruit powder and marjoram powder was reported to mitigate some of these effects [37]. These findings further support the importance of OTA surveillance and the development of effective mitigation strategies to reduce potential adverse effects on animal health.

The remaining mycotoxins showed substantially lower incidence, although their toxicological relevance supports continued monitoring. Patulin (16 notifications), classified by IARC as Group 3 and mainly associated with fruits and apple-derived products, has been linked to genotoxic potential and gastrointestinal effects [38,39]. Zearalenone (3 notifications), known for its estrogenic activity, may act as an endocrine disruptor [40,41]. Fumonisins (26 notifications), primarily detected in cereals, are associated with hepatotoxic and nephrotoxic effects [42]. Deoxynivalenol (13 notifications) and T-2 toxin (7 notifications), mainly identified in cereals and feed, are known for their immunotoxic and cytotoxic properties [43]. In addition, *Claviceps purpurea* (27 notifications) was mainly associated with cereals, posing a risk due to the presence of ergot alkaloids. Overall, although these mycotoxins are less frequently reported, their occurrence highlights the importance of maintaining surveillance across a broad range of compounds, particularly in raw materials intended for both human consumption and animal feed (Table 2).

### 2.6. Food Contaminated by Mycotoxins and Incidence by Food Matrix

#### 2.6.1. Food vs. Feed

The analysis of the dataset revealed a marked disparity between products intended for human consumption and those intended for animal feed. During the study period (2020–2024), a total of 2385 notifications were recorded for food, compared with only 94 for feed, representing 4% of the total notifications. A chi-square goodness-of-fit test showed that the distribution of notifications between food and feed differed significantly from the expected 50:50 distribution (χ^2^ = 2117.26, df = 1, *p* < 0.001), confirming the marked predominance of food-related notifications. Among feed-related notifications, 78 were associated with aflatoxins and 8 with rye ergot (ergot alkaloids), both regulated under Directive 2002/32/EC of the European Parliament and of the Council on undesirable substances in animal feed [44].

However, the relatively low number of feed notifications does not appear to reflect the broader occurrence of mycotoxin contamination in feed. Several studies have shown that mycotoxin contamination in feed is widespread and recurrent, with important implications for the food chain [45,46]. In a large-scale study including 74,821 feed and raw material samples collected between 2008 and 2017 from 100 countries, Gruber-Dorninger et al. [46] reported that 88% of samples were contaminated with at least one mycotoxin, with frequent co-contamination. These findings indicate that mycotoxins are commonly present in feed, although contamination does not necessarily imply non-compliance with regulatory limits and therefore may not result in a RASFF notification. This interpretation is supported by previous studies showing that commercial animal feeds may comply with current European regulatory limits despite the widespread occurrence of mycotoxins, emphasizing that regulatory compliance does not necessarily preclude potential risks associated with long-term, low-dose exposure [47]. Similar trends have been observed in other studies. For example, Yang et al. [45] reported high prevalence rates of DON (91%), zearalenone (70%), aflatoxins (58%), and fumonisins (50%) in swine feed in Taiwan. These data suggest that, despite the low number of feed notifications in RASFF, contamination may be widespread and may represent a relevant long-term risk for both animal health and food safety [45]. These occurrence studies and RASFF notifications should nevertheless be interpreted as complementary rather than directly comparable sources of information, since occurrence surveys assess contamination according to specific sampling designs and analytical methods, whereas RASFF records reflect detected regulatory non-compliances.

The observed disparity between food and feed notifications may be explained by several factors. First, differences in the intensity and focus of official control programmes may influence the probability of detection and reporting. Second, maximum permitted levels for mycotoxins differ between food and feed, which may reduce the number of reported non-compliances in feed despite the frequent presence of these contaminants. In addition, the number of notifications alone does not necessarily reflect the quantity of product potentially affected. Feed consignments may involve substantially different lot sizes and official sampling schemes from those applied to foods intended for human consumption. However, information on the actual batch size corresponding to each individual iRASFF notification was not available in the present dataset; therefore, the volume of feed represented by each notification could not be quantitatively assessed. This should be considered when comparing the apparent impact of food and feed notifications.

From a One Health perspective, the toxicological relevance of feed contamination extends beyond direct effects on animal health and productivity, because certain mycotoxins or their metabolites may enter the human food chain through carry-over processes. A well-established example is the biotransformation of AFB1 ingested by lactating animals into AFM1, which may subsequently be excreted in milk [4,5]. Thus, feed represents a critical entry point for mycotoxins into the food chain, and the comparatively low number of feed notifications should not be interpreted as evidence of negligible risk.

#### 2.6.2. Food Contaminated by Mycotoxins

Nuts represented the food group with the highest incidence of mycotoxin notifications, accounting for 54% of the total. Within this category, aflatoxins were responsible for 98% of the notifications, followed by OTA (2%), fumonisins (<1%), and *Claviceps purpurea* (<1%). This distribution is consistent with the high susceptibility of nuts to fungal growth during cultivation, drying, and storage, particularly under conditions of elevated temperature and humidity. These findings agree with previous analyses of RASFF notifications, which identified nuts as the food group most frequently associated with mycotoxin contamination [15], and with the most recent study by Owolabi et al. [20], who identified nuts as the food group most frequently associated with aflatoxin contamination in RASFF notifications between 2011 and 2021. Fruits and vegetables accounted for 23% of the notifications, with a predominance of aflatoxins (69%) and OTA (32%). In some cases, co-occurrence of aflatoxins and OTA was observed (17 notifications), which explains why the sum of percentages exceeds 100%. Given that these products are widely consumed as part of the daily diet, their involvement is particularly relevant in terms of potential chronic dietary exposure to low concentrations of mycotoxins. Cereals represented the third most affected food group, with 57% of notifications attributed to aflatoxins and 25% to OTA within this category. These results are consistent with previous findings [20], highlighting cereals as a key matrix for mycotoxin contamination. Considering that cereals and cereal-based products are staple foods in many populations, their recurrent involvement reinforces the need for continuous monitoring and effective control strategies at both pre- and post-harvest stages. In contrast, food categories such as wine, ice cream, desserts, and food additives showed minimal incidence (one notification each), likely due to processing conditions and storage characteristics that limit fungal development. This suggests a limited contribution of these matrices to overall dietary exposure to mycotoxins in the general population.

### 2.7. Risk Decisions

The analysis of RASFF notifications for the period 2020–2024 revealed a marked concentration in the “serious” category, with 2279 records (92% of the total). This predominance was consistent across all five years analyzed, ranging from a minimum of 368 cases in 2023 to a maximum of 587 in 2024. In all years, the proportion of notifications classified as “serious” exceeded 85%, reaching values close to 95% in 2021 and 2022. Intermediate and lower-severity categories showed a substantially lower incidence. The category “potentially serious” accounted for 91 notifications (4% of the total), while “potential risk” and “not serious” represented 37 (1.5%) and 19 (1%) notifications, respectively. The “no risk” category was marginal, with only six cases reported over the five-year period. This distribution indicates that most mycotoxin-related incidents were classified within the highest risk levels defined by the RASFF system. The predominance of notifications classified as “serious” in the present study is consistent with previous RASFF analyses which reported that 75.42% of mycotoxin notifications for organic products were also classified as serious, highlighting the significant public health concern posed by these contaminants [31]. Between 2020 and 2022, a limited number of notifications were classified as “undecided”. This may reflect delays in the availability or consolidation of information required for risk categorization; however, the present study does not allow identification of the specific underlying causes. The absence of this category from 2023 onwards suggests improved data completeness and/or greater consistency in classification criteria within the system.

From a toxicological perspective, the high proportion of notifications classified as “serious” indicates that detected concentrations likely exceeded regulatory limits or were associated with exposure scenarios considered relevant by competent authorities. This is particularly important in the case of mycotoxins such as aflatoxins, which are characterized by genotoxic and carcinogenic properties. These findings reinforce the relevance of mycotoxins in chemical risk assessment and public health protection.

It should be noted that RASFF notifications reflect surveillance and enforcement activities rather than the true prevalence of mycotoxins in the food supply. Therefore, they are influenced by differences in sampling intensity, control strategies, and trade flows among countries. As a result, notifications should be interpreted as indicators of regulatory detection rather than direct measures of population exposure.

## 3. Conclusions

The analysis of mycotoxin notifications registered through the iRASFF system during the period 2020–2024, contextualized with general trends since 2012, confirms that mycotoxins remain a persistent challenge for European food safety, although no statistically significant long-term increasing trend was observed. Aflatoxins were the predominant mycotoxins, while nuts, nut products and seeds, and fruit and vegetables were the most frequently affected food categories; most notifications involved products originating from non-EU countries, and border rejections accounted for a substantial proportion of reported cases, with more than 85% of notifications classified as serious throughout the study period. The additional analysis of mycotoxin profiles according to country of origin also revealed differences in the distribution of mycotoxins among geographical regions, with aflatoxins predominating in non-EU products, while EU products showed a more heterogeneous profile. These findings highlight the importance of border surveillance, international cooperation, and risk-based monitoring in limiting dietary exposure to mycotoxins, particularly in globally traded commodities. However, iRASFF data represent detected and reported regulatory non-compliances rather than direct estimates of contamination prevalence, and notification patterns may be influenced by trade volumes, sampling intensity, and national control strategies. In addition, the lack of information on the actual batch size associated with each notification prevents a quantitative comparison of the volume of food and feed potentially affected by individual notifications. Future efforts should integrate analytical surveillance, preventive practices, One Health approaches, and international regulatory cooperation to further reduce chronic exposure and adapt food-safety strategies to evolving scientific evidence, climatic conditions, and global trade dynamics.

## 4. Materials and Methods

To investigate the evolution of alerts related to mycotoxin contamination in food and feed during the period 2020–2024, a systematic active search was conducted using the iRASFF platform, which has been operational since 2020. The dataset included notifications recorded from 1 January 2020 to 31 December 2024 and was retrieved after the end of the 2024 calendar year. In addition, annual RASFF reports published by the European Commission for the period 2012–2019 were consulted in order to provide a historical overview of temporal trends in mycotoxin notifications. These annual reports were not used as the source of the detailed notification data analyzed for the 2020–2024 period.

For data management, a Microsoft Excel database was developed to record information obtained from the iRASFF platform (https://webgate.ec.europa.eu/rasff-window/screen/search, accessed on 21 August 2026), covering the period from 1 January 2020 to 31 December 2024. Only notifications specifically referring to the presence of mycotoxins in food and feed were included, while incomplete records were excluded. Data were filtered according to notification date, type of mycotoxin, type of food product, country of origin, notifying country, risk decision, and notification category. This approach allowed the analysis to be structured from a regulatory surveillance perspective aimed at identifying patterns of occurrence and their potential implications in terms of dietary exposure.

The types of notifications considered in this study included: alert notifications, when a product on the market posed a serious risk to human health and required immediate action; information notifications for attention, when a risk was identified but the product was not present on the market of the notifying country; border rejections, when consignments were rejected at the external borders of the European Union; and information notifications for follow-up, in cases requiring verification of additional corrective measures.

In addition, a narrative literature search was conducted to provide scientific and toxicological context for the interpretation of the iRASFF findings, using PubMed and Web of Science and employing the following keywords: mycotoxins, aflatoxin, zearalenone, ochratoxin, patulin, and food safety. Institutional and regulatory sources, including the Spanish Agency for Food Safety and Nutrition (AESAN), the International Agency for Research on Cancer (IARC), and current European legislation on maximum levels of mycotoxins, were consulted exclusively to support the scientific, toxicological, and regulatory interpretation of the findings. These sources were not used to obtain country-specific notification data, which were retrieved exclusively from the iRASFF database.

### Data Analysis

In a first stage, a descriptive analysis was performed to determine the frequency of notifications according to the type of mycotoxin, affected product category, notifying country, notification category, and country of origin. In a second stage, temporal trend analysis was conducted to identify variations in the number of notifications over the study period and to establish patterns relevant for the interpretation of the results. In addition, potential associations between categorical variables, particularly geographical origin and type of mycotoxin, were evaluated using comparative proportion analyses.

The statistical analysis included the calculation of measures of central tendency (mean) and dispersion (variance and standard deviation), as well as frequency distributions for the variables considered. Inferential statistical analyses were then applied to assess the existence of significant differences between groups. Depending on the number of categories compared, Student’s *t*-test (for two groups) or one-way analysis of variance (ANOVA) (for more than two groups) was used, with a significance level set at *p* < 0.05.

To evaluate the association between categorical variables (year of notification and type of mycotoxin), a chi-square (χ^2^) test of independence was applied. Given the large sample size, Cramér’s V statistic was additionally calculated to estimate the strength of the association. Furthermore, a chi-square (χ^2^) goodness-of-fit test was performed to assess whether the distribution of notifications across different mycotoxins deviated from a uniform distribution, which was considered as the null hypothesis.

All data processing and statistical analyses were performed using Microsoft Excel (version 16.110.3 (26070318)). Prior to the application of parametric tests, the assumptions of normality and homogeneity of variances were verified where appropriate.

## Figures and Tables

**Table 1:** Non-EU countries of origin of products contaminated with mycotoxins (top contributors and aggregated others).

Country of Origin	Number of Notifications	% of Total Extra-EU Notifications
Turkey	585	26.09
United States	353	15.74
India	180	8.02
Egypt	171	7.63
Iran	155	6.91
Argentina	146	6.51
Pakistan	127	5.75
China	63	2.81
Georgia	63	2.81
Indonesia	46	2.05
Other countries (*n* = 35)	353	15.74
Total	2242	100

**Table 2:** Distribution of the various mycotoxin-related notifications in food products.

	Total		Aflatoxin		Ochratoxin		Fumonisin		Other Mycotoxins	
Type of Product	Count	%	Count	%	Count	%	Count	%	Count	%
Cereals and bakery products	255	10.29	146	7.03	65	18.01	22	84.62	32	48.48
Cocoa and cocoa preparations, coffee and tea	16	0.65	3	0.14	14	3.88	0	0.00	0	0.00
Compound feeds	3	0.12	2	0.10	0	0.00	1	3.85	1	1.52
Confectionery	5	0.20	5	0.24	0	0.00	0	0.00	0	0.00
Dietetic foods, food supplements and fortified foods	3	0.12	5	0.24	1	0.28	0	0.00	1	1.52
Feed materials	73	2.94	61	2.94	0	0.00	1	3.85	12	18.18
Food additives and flavourings	1	0.04	0	0.00	0	0.00	0	0.00	1	1.52
Fruits and vegetables	577	23.28	400	19.27	187	51.80	0	0.00	7	10.61
Herbs and spices	149	6.01	106	5.11	48	13.30	0	0.00	0	0.00
Ices and desserts	1	0.04	1	0.05	0	0.00	0	0.00	0	0.00
Milk and milk products	3	0.12	3	0.14	0	0.00	0	0.00	0	0.00
Non-alcoholic beverages	7	0.28	0	0.00	0	0.00	0	0.00	7	10.61
Nuts, nut products and seeds	1346	54.30	1322	63.68	30	8.31	2	7.69	1	1.52
Other food product/mixed	25	1.01	17	0.82	9	2.49	0	0.00	1	1.52
Prepared dishes and snacks	7	0.28	2	0.10	2	0.55	0	0.00	3	4.55
Soups, broths, sauces and condiments	7	0.28	3	0.14	4	1.11	0	0.00	0	0.00
Wine	1	0.04	0	0.00	1	0.28	0	0.00	0	0.00
Total	2479	100.00	2076	100.00	361	100.00	26	100.00	66	100

Note: Percentages are calculated within each mycotoxin group and represent the proportion of notifications corresponding to each product category. Mycotoxin categories are not mutually exclusive, as a single notification may report the simultaneous presence of multiple mycotoxins; therefore, percentages across mycotoxin groups within a product category are not expected to sum to 100%. For the “Other mycotoxins” category, percentages are calculated using the total number of notifications included in that group as the denominator; consequently, the percentages across product categories sum to 100%.

## Data Availability

The original contributions presented in this study are included in the article. Further inquiries can be directed to the corresponding author.
